# Moisture-Responsive Graphene Actuators Prepared by Two-Beam Laser Interference of Graphene Oxide Paper

**DOI:** 10.3389/fchem.2019.00464

**Published:** 2019-06-27

**Authors:** Hao-Bo Jiang, Yan Liu, Juan Liu, Shu-Yi Li, Yun-Yun Song, Dong-Dong Han, Lu-Quan Ren

**Affiliations:** ^1^Key Laboratory of Bionic Engineering (Ministry of Education), Jilin University, Changchun, China; ^2^State Key Laboratory of Integrated Optoelectronics, College of Electronic Science and Engineering, Jilin University, Changchun, China

**Keywords:** graphene oxide paper, two-beam laser interference, micronanostructure, bilayer structure, moisture-responsive actuator

## Abstract

Here, we reported an ingenious fabrication of moisture responsive graphene-based actuator via unilateral two-beam laser interference (TBLI) treatment of graphene oxide (GO) papers. TBLI technique has been recognized as a representative photoreduction and patterning strategy for hierarchical structuring of GO. The GO paper can be reduced and cut into grating-like periodic reduced graphene oxide (RGO) microstructures due to laser ablation effect. However, the lower light transmittance of the thick GO paper and the corresponding thermal relaxation phenomenon make it impossible to trigger complete reduction, leading to the formation of the anisotropic GO/reduced GO (RGO) bilayer structure. Interestingly, the RGO side that feature lower OCGs and higher roughness shows strong water adsorption due to the formation of micronanostructures. Due to the different water adsorption capacities of the two sides, a flower moisture-responsive actuator has been fabricated, which exhibits “opening” and “closing” behavior under different humidity conditions.

## Introduction

The traditional actuators, such as energy conversion devices, sensors, robotics, and micro-electromechanical systems, are driving devices which have been extensively studied because of their irreplaceable application in basic scientific research and engineering technologies (Azizi and Khorasani, 2011; Petit et al., 2012; Tottori et al., 2012). However, with the emergence and development of technology industries, next-generation intelligent products without additional connections, and energy-supply equipment, such as wearable electro-skins (Ho, 2002), artificial muscles (Han et al., 2019), microrobots in tissue engineering (Kim et al., 2012), and lab-on-a-chip (LoC) systems (Kokalj et al., 2014), are in increasing demand. For this current situation and future prospects, stimuli-responsive materials (SRMs) that can rapidly and reversibly convert their structure/morphology/volume variation into mechanical deformation under certain environmental stimulation (Deng et al., 2018; Han B. et al., 2018) are ideal choices. External stimuli including light, temperature, electromagnetism, solvents, and humidity can be utilized for realizing intelligent actuators (Tian et al., 2011; Kim et al., 2018; Li and Yin, 2019; Zhu et al., 2019). As a typical example, an anisotropic bilayer structure can express the mechanical work generated in different material layers by external stimulus more efficiently and intuitively (Ma et al., 2018; Ohuchi et al., 2018). Many research groups have made successful progress in the study of bilayer actuators. For instance, Zhang et al. investigated the controlling helicity angle, chirality, diameter, and pitch of multilayered SiGe/Si/Cr nanobelts with widths <400 nm (Zhang et al., 2006). Kelby et al. presented an Au-polymer bilayer brush example for the controlled folding of 3D micro-structures (Kelby et al., 2011). However, as moving deformation occurs step by step in a motion device, poor adhesion between the different material layers directly affects the mechanical strength of the device during movement (Xin et al., 2016; Rodrigo et al., 2017; Padhi et al., 2018). In this regard, it is already a challenge to exploit new monomer material to design and fabricate smart bilayer-structure actuator through the precise control of the unilateral morphology and composition, which could realize the anisotropic property.

Graphene oxides (GOs), as derivatives of graphene materials (Zhu et al., 2010), possess immense potential for a broad range of graphene-device applications owning to several advantages, such as solubility, batch preparation, ease of fabrication of large films, eco-friendliness, and stable physicochemical properties (Becerril et al., 2008; Eda et al., 2008; Kudin et al., 2008). As the surface contains numerous hydrophilic oxygen-containing groups (OCGs), desiccative GO films are extremely sensitive to ambient humidity (Han D. et al., 2018). In spite of surface defects of GO, suitable chemical, thermal and photoreduction method have been successfully developed to realize some sort of OCGs removal, which leads to the formation of RGO structure with different components from GO (Stankovich et al., 2007; Li et al., 2011). In view of this principle, graphene smart actuators based on GO material have been successfully developed. Qu et al. successfully prepared the fiber-type smart robots through controllable laser reduction of GO fibers, which have been realized a series of one-dimensional mechanical deformations, such as folding, bending, and S-shaping (Cheng et al., 2013). Zhang et al. presented a facile preparation of moisture responsive graphene actuators by unilateral UV irradiation of graphene oxide (GO) papers, which have been mimicked the cilia of respiratory tract and tendril climber plant to transport objects (Han et al., 2015a). However, although the partly photoreduction treatment endow stimuli-responsive properties to the GO/RGO structure, the above-mentioned works with respect to regulation of the chemical compositions of the materials weaken the structure/morphology influence. It is blank to fabricate such graphene bilayer structure. The unique micronanostructures of material surfaces have attracted interest in a wide range of scientific fields, such as electronic devices, energy storage devices and biomimetic surfaces (Bi et al., 2013; Kumar et al., 2014; Wang et al., 2018), which will hold great potential in the field of smart actuators.

Herein, inspired by biomimetic structural surfaces, a micronano-patterning photoreduction method has been applied to prepare moisture responsive GO/RGO bilayer structure through the laser holography technique treatment of GO paper. The chemical properties of the GO surface can be considerably altered after two-beam laser interference (TBLI) treatment by the photothermal effects, causing the removal of most of the OCGs on and between the GO sheets. Simultaneously, abundant RGO micronanostructures including periodic micro-gratings and nanoscale sheets are formed, increasing the richness of the surface morphology, this changes the wettability of the resultant graphene films, causing hydrophobicity. Laser processing technology is an effective means to realize controlling of surface wettability by fabricating micro/nano structures on the surface of materials (Yin et al., 2017b, 2018; Duan et al., 2018), which own great potential in application of superhydrophilicity (Yin et al., 2017a; Yang et al., 2018). Considering the limited light transmittance of GO paper and thermal relaxation could not realize the totally reduction of GO paper, a gradient photoreduction process along the sectional direction of GO paper occurs spontaneously. Consequently, the anisotropic GO/RGO bilayer structure has been fabricated during the self-acting photoreduction. Taking advantage of the different water-absorbing capacities of the two sides of bilayer structure, the resultant GO/RGO film could be acted as a moisture-responsive actuator. We further demonstrated the graphene actuator to mimic opening and closing behavior of a flower.

## Materials and Methods

### Preparation of GO Paper

The graphene oxide material we used here was prepared by Hummer's method. After high strong oxidant (such as H_2_SO_4_, KMnO_4_ and so on) treatment, the graphite powder was oxidized into single-layer and few-layer graphene nanosheets containing a large number of hydroxyl and carboxyl groups. Due to the presence of a mass of OCGs, the obtained GO material is easily soluble in water and becomes a water-soluble suspension. In order to remove residual sulfate and chloride ions, GO aqueous solution should be washed several times with deionized water. The synthesized GO solution was collected by centrifugation and then dispersed in distilled water at a concentration of 3 mg/mL under ultrasonic treatment. Subsequently, graphene oxide paper has been fabricated by ultrafiltration of the prepared GO aqueous solution with the help of the filtration membrane with pore diameter of 0.22 μm. And drying the GO/membrane in the ambient air, and then peeling off the GO paper for next treatment.

### TBLI Reduction of GO Paper

A standard single-mode Nd:YAG laser (Spectra-Physics, 355 nm, 10 Hz, and 10 ns pulse duration) was used as the light source for the GO reduction process. The laser beam with the diameter size of 8 mm was split into two beams, which own the same optical path lengths to the sample surface. The RGO with multilevel structures were fabricated by exposing the GO papers to the interfered laser region. Taking advantage of laser holography technique, the periodicity of the grating could be precisely controlled by changing the angle of two laser beams. By adjusting the appropriate laser power (~0.4 mW), exposure time (30 s) and laser light path, here we fabricated the graphene grating with 2 μm. The laser power is 0.4 mw, and the exposure time is 30 s, which is the relatively suitable laser processing parameter. When the laser power is too large and the exposure time is too long, the surface morphology of the processed sample will be ablated seriously. On the contrary, when the laser power is too low and the exposure time is too short, it is difficult to form a complete continuous periodic micro-nano grating structure (Supplementary Figure S3). Taking advantage of laser holography technique, the periodicity of the grating could be precisely controlled by changing the angle of two laser beams, as shown in the following equation:

$$\begin{array}{l} \Lambda =\frac{\lambda_{F}}{2\sin\left(\theta /2\right)} \end{array}$$

where Λ is the period, λF is the laser wavelength and θ is the angle between two beams.

## Results and Discussion

### Characterization

SEM images were obtained using a field-emission scanning electron microscope (JSM-7500, JEOL, Japan), which referred to as FE-SEM. X-ray photoelectron spectroscopy (XPS) was performed using an ESCALAB 250 spectrometer for chemical bond analysis. Raman spectroscopy was recorded on a Jobin-Yvon T64000 Raman spectrometer equipped with a liquid-nitrogen-cooled argon ion laser at 514.5 nm (Spectra-Physics Stabilite 2017) as the excitation source; the laser power used was ~10 mW with an average spot size of 1 μm in diameter. The contact angle (CA) measurements were performed using the Contact Angle System OCA 20 (DataPhysics Instruments GmbH, Germany) at ambient temperature. The CA was measured using a 4 μL water droplet. The humidity response cycle test is carried out in an environment with 100% humidity. The controlled humidity environments were achieved using saturated aqueous solutions of CH_3_COOK, MgCl_2_, K_2_CO_3_, NaBr, NaCl, and KCl in a closed glass vessel, which yielded ~23, 33, 44, 57, 75, and 86% RH, respectively. All of the measurements were conducted in air at room temperature (25°C).

### Moisture Response of GO Paper

**Figure 1:**
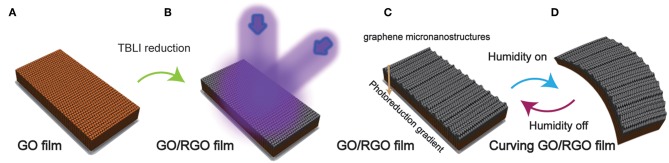
The schematic illustration of the fabrication process of a moisture responsive GO/RGO bilayer film. **(A)** GO film. **(B)** TBLI treatment of GO film. **(C)** GO/RGO film. **(D)** Curving GO/RGO film.

As an efficient tool for large-area micronano-patterning without any shadow masks and chemicals, laser holography technique is undoubtedly a preferred choice for fabrication of micronanostructures (Wang et al., 2011; Sun et al., 2018). As a typical example, TBLI has already proved its value in the photoreduction of GO and fabrication of graphene micronanostructures synchronously in our previous works (Jiang et al., 2014). Hence, in this study, we applied the TBLI as the irradiation source to prepare RGO structure and fabricate the GO/RGO bilayer structure, for realizing humidity response. Figure 1 displays a schematic illustration of the design philosophy and manufacturing flow of moisture-responsive graphene actuator. We use the GO papers as experimental materials, which have been fabricated by vacuum filtration of GO aqueous through a filter membrane and naturally dried in air. The yellow-brown dried GO papers are showed in Figure 1A. Subsequently, after the GO paper was exposed to the laser interference region, the RGO side turned black and it may result from the removing of OCGs (Figure 1B). During the laser machining process, the periodic micro-gratings with nanoscale roughness have been produced along the direction of laser intensity distribution. In the laser interference region, the laser intensity distribution is constant along the y-axis, and sinusoidal along x-axis. The highest is four times of each laser beam, and the lowest is zero. At the high laser intensity region, the OCGs were thoroughly removed; whereas at the low intensity region, the GO survived, and was reduced partially. However, due to the limited light transmission of GO paper and delayed thermal transmission, the reduction reaction on the GO paper surface was incomplete. In this way, anisotropic GO/RGO bilayer structure has been realized along the sectional direction of GO paper, which showed in Figure 1C. For plane RGO surface, the hydrophilic GO surface has a stronger water absorbing capacity compared to the RGO because of the formation of hydrogen bonds on the GO sheets in the presence of moisture, as reported previously (Han et al., 2015b). But TBLI photoreduction method induced increased interlayer space and the new products of graphene micronanostructures on the RGO surface, such morphological changes would provide more space for water molecules to interact with RGO sheets through Van der Waals force. On account of this situation, when the GO/RGO bilayer film was exposed to humid environment, it would bend toward the GO side due to the asymmetric adsorption, as shown in Figure 1D.

**Figure 2:**
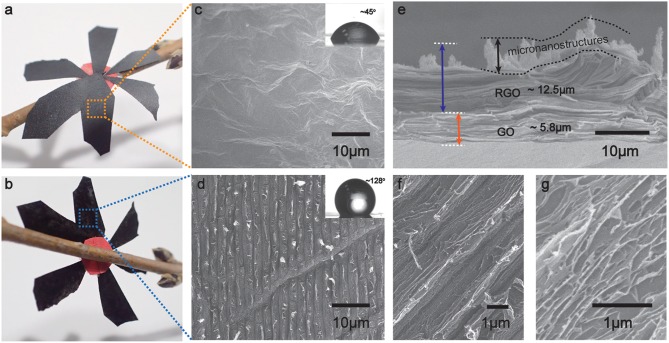
**(A,B)** Photographs of the resultant GO/RGO bilayer structure viewed from the front side **(A)** and the reverse side **(B)**, respectively. **(C–G)** SEM images of GO/RGO bilayer structure. **(C,D)** SEM images of GO side and RGO side. The insets show water droplet contact angles of both sides. **(E)** SEM image of the sectional view of GO/RGO bilayer structure. **(F,G)** High-resolution SEM images of RGO side.

As observed in the optical micrographs of bilayer structure (Figures 2A,B), the resultant RGO paper presents black in comparison with the dark brown of GO, proving the effective photoreduction of GO. However, the different color behaviors of two sides of the sample reveal the gradient change of the photoreduction process. The maximum diameter of light spot is about 1 cm, which should be taken 30 s for the fabrication of each spot with the grating structures. Large area of RGO structure surface can be prepared by splicing each spot together. The area has no upper limit. In order to investigate the TBLI photoreduction process, scanning electron microscopy (SEM) images of the GO and RGO sides were obtained. Figure 2C shows the SEM image of relatively smooth GO surface with rich wrinkles owned by unique graphene materials. Due to the laser ablation, ordered grating structures with a period of 2 μm were fabricated. Despite these periodic micro-gratings, the folded structures of graphene could be observed on the RGO side. To characterize the surface wettability of the two sides of bilayer structure, static water-droplet contact angle (CA) measurements have been applied, which could be observed in the insets of Figures 2C,D. The CA value of the GO side was ~45°, whereas the RGO side gives a significantly increased CA of ~128°, which is a significant improvement compared to the UV and other reduction methods. Generally, the obvious hydrophobicity on RGO surface could be mainly put down to the drastic removal of OCGs and preparation of graphene micronanostructures during photoreduction. It is to be noted that the CA of the RGO surface would show a certain descending over time because of the permeation of water droplets on the surface of GO paper.

Next, a section-view SEM image of the TBLI treated GO paper has been presented. In Figure 2E, the fluffy structures with larger layer-gaps (thickness is ~12.5 μm) in the up-side and the relatively dense structures with thinner thickness of ~5.8 μm in the bottom-side could be observed. Such formation of asymmetric structure is ascribed to the result of gas release, such as CO_2_, CO, H_2_O in the reduction layer, which have been produced by carbon components in the photothermal reaction. In the case of this situation, the thicknesses of the two layers structure show certain dependence on sectional dimension of GO paper and laser intensity of the TBLI treatment. Thus, we could observe a layer of micronanostructures on the top of up-side. To get further insight into the detailed morphology of micro-gratings, we measured the magnified SEM images of RGO surface (Figures 2F,G). Laser cutting effect not only prompts the formation of periodic micro-scale grating-like structures but also in the meantime induces the graphene nano-layer structures. The special attention should be paid to the laser parameters and optical path design. They will affect the structural morphology of the RGO surface. Adjusting the intensity and exposure time with high level, the GO paper could realize violent reduction or even combustion.

**Figure 3:**
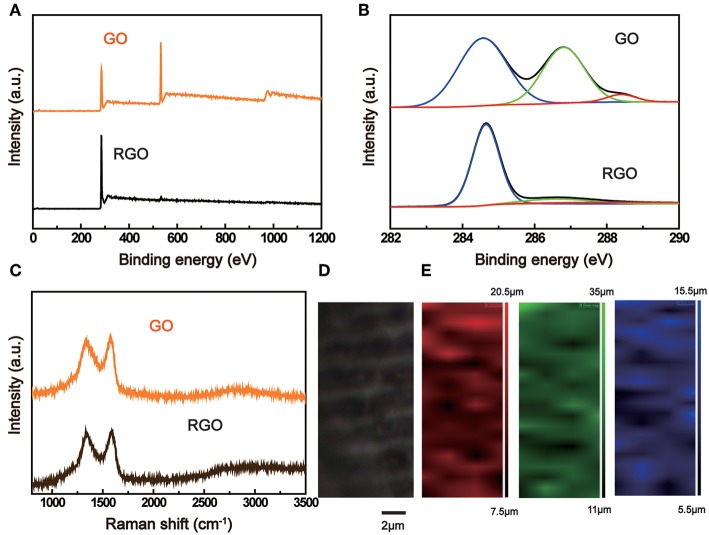
**(A)** Survey XPS spectra and **(B)** C1s spectra of GO/RGO film measured on the GO side and RGO side. **(C)** Raman spectra of GO/RGO structure measured on the GO side and RGO side. **(D)** Optical image of RGO structure. **(E)** Raman mapping of **(D)**, corresponding to wave number from 1,300–1,500, 1,500–1,800, 2,200–2,800 cm^−1^, respectively.

To evaluate the chemical composition change, we measured the X-ray photoelectron spectroscopy (XPS) of the two sides of the RGO paper irradiated by TBLI light. As shown in Figure 3A, the carbon and oxygen content comparisons between GO and RGO side are surveyed. The C/O ratio of the RGO side shows an increased value of 35.76 and the GO is about 2.52. This obvious change reveals the photoreduction gradient of GO/RGO bilayer structure. Besides, the C1s spectra of GO and RGO sides could be split into three peaks that correspond to C-C (284.6 eV, non-oxygen ring), C-O (286.8 eV, hydroxyl and epoxy carbon), and C = O (288.4 eV, carbonyl), as shown in Figure 3B. Obviously, OCGs are very rich on the GO surface, the C-O and C = O peaks of GO side show an higher value than that of RGO side irradiated after TBLI laser. And the C content increased from 71.58 to 97.28%, indicating the removal of OCGs. X-ray diffraction (XRD) of GO and RGO side have also been tested (Supplementary Figure S1). The XRD pattern of the GO sheets shows a typical diffraction peaks at 2θ = 11.55°, which indicates an ordered layered structure of GO. However, after TBLI treatment, the diffraction peak disappeared, which suggests that the RGO layers become disorder after the removal of OCGs.

Furthermore, another characterization method, Raman spectroscopy has been used to test the degree of hybridization of GO and RGO, as shown in Figure 3C. Two characteristic peaks at 1,343 cm^−1^ (D band) and 1,588 cm^−1^ (G band) are observed. The G peak is used to characterize the degree of graphitization of carbon materials, and the D peak represents the defects, caused by dislocation, grain boundary, fold, and so on. In general, after the removal of OCGs by appropriate reduction method, the C-C hybridization on the surface would restore on a certain extent, and I_D_/I_G_ shows an obvious decreased. However, the I_D_/I_G_ ratio of RGO in our work slight increased from 0.97 (GO) to 0.98, which means that more new defects have been brought during the laser ablation. To further investigate the uniformity of laser interfered region of RGO, we also measure the Raman maps in an area of 5 × 10 μm^2^, as shown in Figure 3D. The spatial distribution of D, G, and 2D peak values are presented as red, green and blue maps, respectively (Figure 3E). It could be clearly observed that the D, G band intensity distribution almost keep the same as its morphology in optical microscope, which indicates chemical composition of the structures obtained by TBLI treatment was also relatively homogeneous.

**Figure 4:**
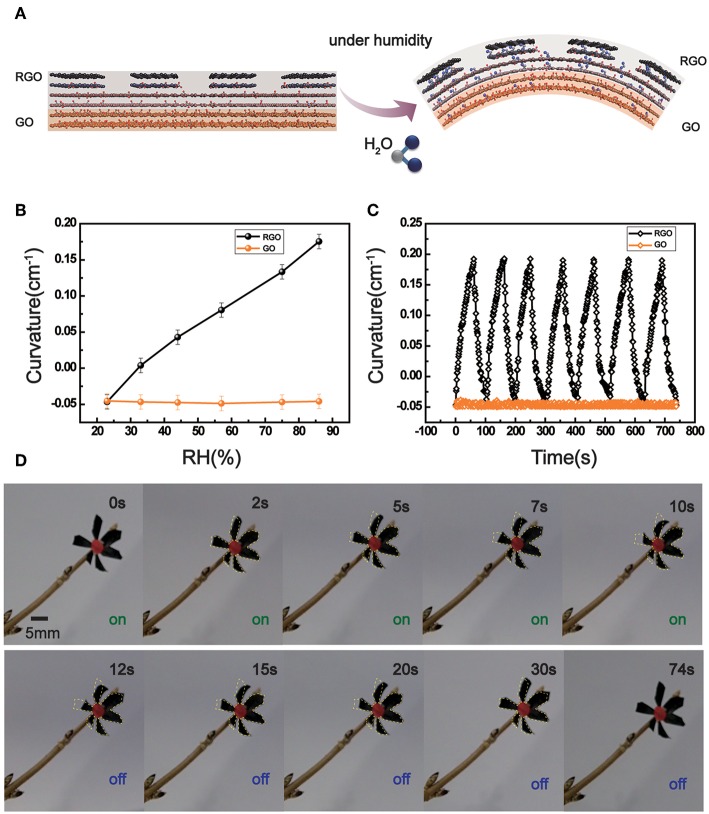
**(A)** Schematic illustration of the bending behavior of the GO/RGO bilayer structure. Water molecules have been demonstrated in blue color in GO/RGO structure for easy to identify. **(B)** Bending curvature for GO paper and GO/RGO bilayer structures. **(C)** Reversible moisture response of GO and GO/RGO. **(D)** The moisture-responsive flower robot made of GO/RGO structure.

After TBLI treatment, the GO/RGO bilayer structure has been formed, which would curve to the absorbent layer in moisture. Figure 4A shows the mechanism of deformation under humidity control. In general, the GO surface is hydrophilic due to lots of OCGs in the interlayer. Since the presence of OCGs accounts for the formation of hydrogen bonding to adsorb water. However, abundant micronanostructures (micro-gratings, nano-layers) have been made during the process of photoreduction on the RGO surface. Even though RGO layer has fewer hydrophilic groups, unique structures provide more space for water molecules to enter and adsorb. Considering the anisotropic water adsorption between two sides, the layer spacing of RGO side would become larger than GO, leading to GO/RGO ribbon bending to the GO side under high humidity. We further test the dependence of the bending curvature and humidity (RH) of the two layers, as shown in Figure 4B. As the humidity gradually increases, the bending degree of the bilayer structure becomes more obvious. When the humidity is 86%, the GO/RGO bilayer paper owns maximum bending angle and the curvature reaches 0.175. The original bilayer structure also has a certain degree of curvature (−0.047). By contrast, the pure GO paper is hard to bend in moisture. When the humidity is “on” and “off,” the bilayer structure exhibits a reversible bending deformation. Figure 4C shows the highly repeatable cycling behavior upon seven cycles. For the GO/RGO bilayer structure, the response and recovery times are ~55 and ~45 s, respectively. Moreover, the GO/RGO bilayer also shows excellent stability during frequent bending–unbending actuation, and the curvature almost keeps a consistent value, indicating the good reproducibility, and precision of the actuation function (Supplementary Figure S2).

The humidity response characteristics of the GO/RGO bilayer structure provide possibilities for the design and fabrication of intelligent graphene actuators. In our work, inspired from opening and closing behaviors of flowers, the unique flower-robot has been demonstrated. We cut the GO/RGO bilayer papers into six pieces to simulate the shape of the petals; and then put them together and glue to a branch directly, as shown in Figure 4D. When the environmental humidity is “on,” the flower robot would exhibit the “closing” behavior. The bottom petals of flower robot were observed not moving in Figure 4D because of video recording angle. It took only 10 s for the “flower” to go from full bloom state to final closure. For comparison, we mark the original state of flowers in yellow. When the moisture is replaced by dry air, the “flower” slowly opens again from the closed state. The recovery process without any external energy supports lasts 64 s. The changes in humidity conditions can operate the GO/RGO bilayer structure, which also can be made into other forms of robots, such as claw, crawler and tendril.

## Conclusions

In conclusion, TBLI treatment of GO papers has been evolved for successful fabrication of GO/RGO bilayer structure toward preparation of moisture-responsive graphene-based actuators. TBLI treatment was performed to remove the OCGs on the GO sheets and form graphene micronanostructures. Due to the light gradient transmittance and delayed thermal transmission of GO paper, the gradual reduction along its lateral direction occurred, resulting in differences in the reduction degree as well as surface morphology on both sides. In spite of hydrophilicity of GO, the RGO side has been observed stronger absorption ability of water molecules than GO side because of auxetic material porosity induced under laser ablation, which rendered the anisotropic GO/RGO structure impressible to environmental moisture. Interestingly, the graphene micronanostructures formed during the reduction process simultaneously increased the roughness of the RGO surface, leading to hydrophobicity on the RGO side. A stripe GO/RGO bilayer structure has been fabricated to demonstrate the reversible bending behaviors. A curvature of −0.047 to 0.175 was obtained, when the RH was adjusted between 23 and 86%. Inspired by the floral behavior, the fabricated humidity-driven graphene actuators were assembled to mimic the opening and closing behavior of flower. We deem that this laser lithography technology may enable biomimetic design and fabrication of intelligent graphene-devices.

## Data Availability

The raw data supporting the conclusions of this manuscript will be made available by the authors, without undue reservation, to any qualified researcher.

## Author Contributions

H-BJ, YL, and L-QR conceived the idea and designed the experiments. H-BJ, S-YL, and Y-YS made the characterizations. H-BJ and JL performed the detections. H-BJ, D-DH, and YL contributed to data analysis and interpretation. H-BJ and D-DH wrote the paper. All authors discussed the results and commented on the manuscript.

### Conflict of Interest Statement

The authors declare that the research was conducted in the absence of any commercial or financial relationships that could be construed as a potential conflict of interest.
