# Supplementary material for: Moisture-Responsive Graphene Actuators Prepared by Two-Beam Laser Interference of Graphene Oxide Paper
Source: Front Chem. 2019 Jun 27;7:464. doi: 10.3389/fchem.2019.00464 (PMC6610323; doi:10.3389/fchem.2019.00464)
Supplement: Supplementary file 1 [file Presentation_1.pdf]

Supporting information for

Moisture-responsive graphene actuators prepared by two-beam laser interference of graphene oxide paper

Hao-Bo Jiang[1], Yan Liu[1], Juan Liu[1], Shu-Yi Li[1], Yun-Yun Song[1], Dong-Dong Han[2]*,*

and Lu-Quan Ren[1]

1. Key Laboratory of Bionic Engineering (Ministry of Education), Jilin University, Changchun, China
2. State Key Laboratory of Integrated Optoelectronics, College of Electronic Science and Engineering, Jilin University, Changchun, Chin

Corresponding Author
*E-mail: lyyw@jlu.edu.cn; handongdong@jlu.edu.cn.

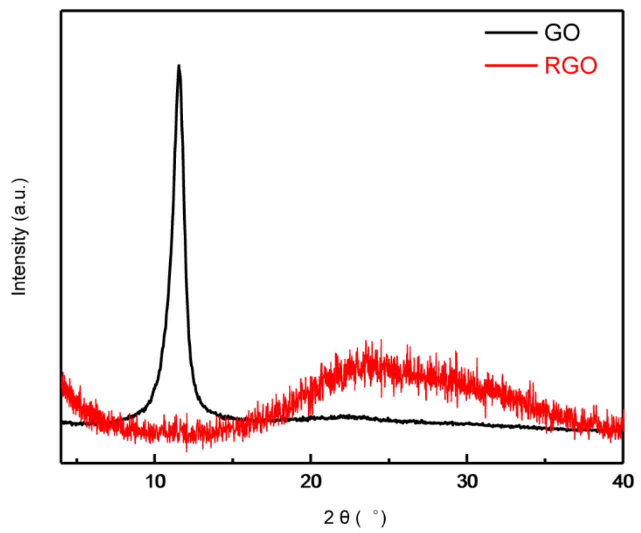

Figure S1. XRD patterns of GO and RGO.

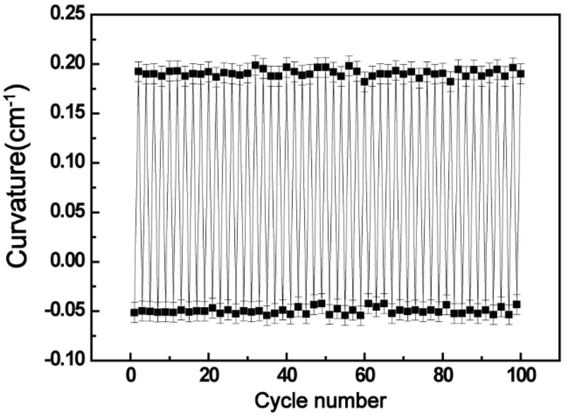

Figure S2. Cycling testing result for GO/RGO bilayer structure.

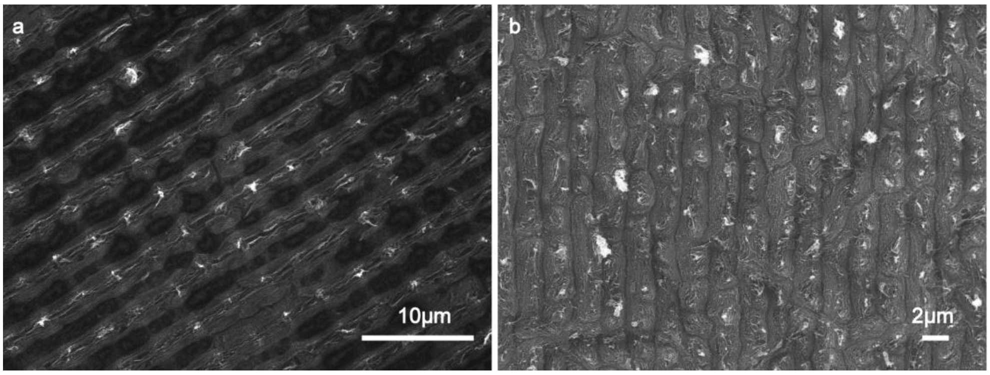

Figure S3. SEM images of RGO structure surface which made under (laser power: 0.8 mW, exposure time: 50s) and (laser power: 0.3 mW, exposure time: 10s) laser processing parameters.
